# Advances in sparse dynamic scanning in spectromicroscopy through compressive sensing

**DOI:** 10.1371/journal.pone.0285057

**Published:** 2023-11-09

**Authors:** George Kourousias, Fulvio Billè, Francesco Guzzi, Matteo Ippoliti, Valentina Bonanni, Alessandra Gianoncelli

**Affiliations:** Elettra–Sincrotrone Trieste, Basovizza, Trieste, Italy; University of Delhi, INDIA

## Abstract

Scanning microscopies and spectroscopies like X-ray Fluorescence (XRF), Scanning Transmission X-ray Microscopy (STXM), and Ptychography are of very high scientific importance as they can be employed in several research fields. Methodology and technology advances aim at analysing larger samples at better resolutions, improved sensitivities and higher acquisition speeds. The frontiers of those advances are in detectors, radiation sources, motors, but also in acquisition and analysis software together with general methodology improvements. We have recently introduced and fully implemented an intelligent scanning methodology based on compressive sensing, on a soft X-ray microscopy beamline. This demonstrated sparse low energy XRF scanning of dynamically chosen regions of interest in combination with STXM, yielding spectroimaging data in the megapixel-range and in shorter timeframes than were previously not feasible. This research has been further developed and has been applied to scientific applications in biology. The developments are mostly in the dynamic triggering decisional mechanism in order to incorporate modern Machine Learning (ML) but also in the suitable integration of the method in the control system, making it available for other beamlines and imaging techniques. On the applications front, the method was previously successfully used on different samples, from lung and ovarian human tissues to plant root sections. This manuscript introduces the latest methodology advances and demonstrates their applications in life and environmental sciences. Lastly, it highlights the auxiliary development of a mobile application, designed to assist the user in the selection of specific regions of interest in an easy way.

## 1. Introduction

Scanning techniques based on the serial acquisition of signals have the fundamental advantage of collecting signal point-by-point in a raster scan, which enables a researcher to simultaneously provide multi-signal information from specific illuminated areas, when multiple types of detectors are installed [1–3]. The obtained spatial resolution depends only on the spot size and the step size. However, scanning methods have the intrinsic drawback of acquiring signals point-by-point in a serial way and can therefore be intrinsically slow, as the overall scan time depends on the number of points and the time required to acquire each point. Moreover, often, for practical reasons mainly related to the precision and the accuracy constraints of the scanning stage, scans are acquired line by line, providing square or rectangular datasets, even when the region of interest may have an irregular shape and may cover overall a much smaller portion of the total scanned area.

The scanning speed can be increased by improved technology, both from the detector and the optics/source side, by providing higher photon flux and more sensitive (and thus faster) and/or larger detectors. On the other hand, collecting data over areas without any real sample (areas where only the sample’s support is present) or in regions of limited interest, still remains an open problem. Scanning such areas of limited interest within the sample has two main drawbacks: i) collecting signal on useless portions of the sample, thus employing the time in an inefficient way and ii) storing data that do not contain relevant scientific information regarding the specimen, thus occupying valuable storage space.

These issues can potentially impact a much broader class of scanning methods, for which faster scans would provide a means to disentangle the experiment’s success from source and/or detector instabilities over time.

Many experiments, including synchrotron beamtimes, would benefit greatly from increased acquisition speeds, mostly due to the high costs associated with operating the facility and the time related to the submission and acceptance of a research proposal. In general, the possibility of making the acquisition more time-efficient, allows a better use of synchrotron beamtime and therefore a higher number of users and/or experiments.

Recently we have proposed a new smart acquisition approach based on the compressive sensing concept [4, 5], which has been demonstrated for soft X-ray microscopy and X-ray Fluorescence approaches.

Compressive sensing or compressed sensing (CS) is a signal processing technique which aims at reconstructing a signal from a series of discrete sampling measurements, even in areas where the signal is not acquired [6]. It is based on sparse representations and it relies on specific assumptions that enable the reconstruction. While conventional approaches are based on the Nyquist-Shannon theorem, where the sampling rate must be at least twice the maximum frequency present in the signal (so-called Nyquist rate), CS approaches can recover certain signals and images from far fewer samples or measurements than the traditional methods [7].

Compressive sensing is an emerging field, first employed in astronomy [8], to extract information from sparse signals, but that can be applied to a wider range of applications and fields. In particular, CS was recently applied to microscopy and spectroscopy, as many imaging and spectroscopic techniques can benefit from it [4, 5, 9–15]. Our group has been the first one to introduce this methodology and demonstrate its potential for X-ray Fluorescence (XRF) microscopy [4, 5]. CS has already been used in clinical imaging, for instance Siemens is proposing it for MRI cardiac and abdominal imaging to speed up the acquisition time, since this exam requires patients to hold their breath [14].

In our previous works [4, 5] we have presented different acquisition methods, where CS can be applied in XRF microscopy in a dynamic way. In particular the decision regarding whether and where to acquire sparse data can be driven by the XRF detector itself, evaluating whether the element of interest is present or absent, or by exploiting auxiliary and faster detectors available in the setup itself [4, 5].

In this paper we report an evolution of the proposed methods, with the aim of making such a smart acquisition approach more user friendly and more robust, and thus more easily available and profitable to synchrotron users and in general to imaging.

## 2. Material and methods

**Fig 1:**
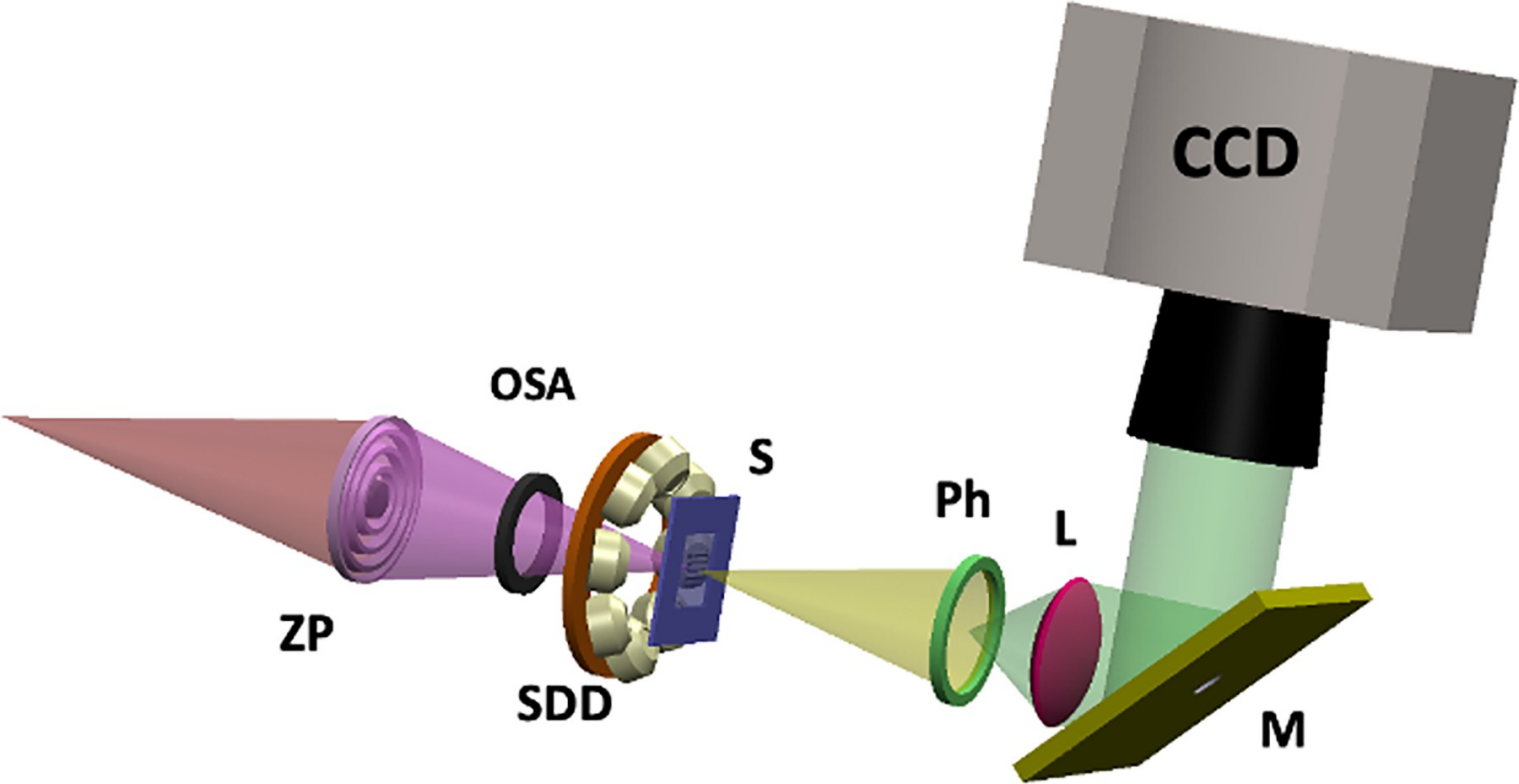
TwinMic STXM mode. Schematic view of the TwinMic STXM mode set up equipped with a microprobe forming zone plate (ZP) on the specimen (S), a diffraction order selecting aperture (OSA), a transmission detection system based on a fast read-out CCD camera (CCD) and a visible light converting system (VLCS), and with a low-energy X-ray fluorescence (LEXRF) detector system consisting of 8 Silicon Drift Detectors (SDDs) in backscattered configuration. The VLCS consists of a Phosphor screen (Ph), a lens (L) and a 45 degrees tilted Mirror (M).

The experiments presented here were carried out at the TwinMic soft X-ray Microscopy beamline [16, 17] of Elettra Sincrotrone Trieste (Trieste, Italy). TwinMic was operated in STXM mode where a 600 μm diameter and 50 nm outermost width zone plate focuses the monochromatised X-ray photons onto the sample, while the latter is raster scanned perpendicularly to the optical axis of the X-ray beam. In this operation mode, the transmitted photons are collected through a fast readout CCD camera (DV860, Andor Technology) point by point during the scan, producing absorption and differential phase contrast images [2]. Simultaneously, the XRF photons emitted by the samples can be collected point by point in the raster scan by 8 Silicon Drift Detectors (SDD) located upstream symmetrically in front of the sample, positioned off axis to prevent shadowing of the CCD [16], generating XRF elemental maps of the area under analysis. Fig 1 reports a sketch of the TwinMic STXM setup.

The idea of developing smart acquisition strategies at TwinMic arose from the fact that at present, XRF scans may take hours to be completed due to: i) mechanical constraints which do not allow a large enough solid angle fraction and ii) to the available energy range (400–2200 eV), which is characterised by low X-ray Fluorescence Yields as the competing Auger phenomenon becomes dominant below 2 keV.

**Fig 2:**
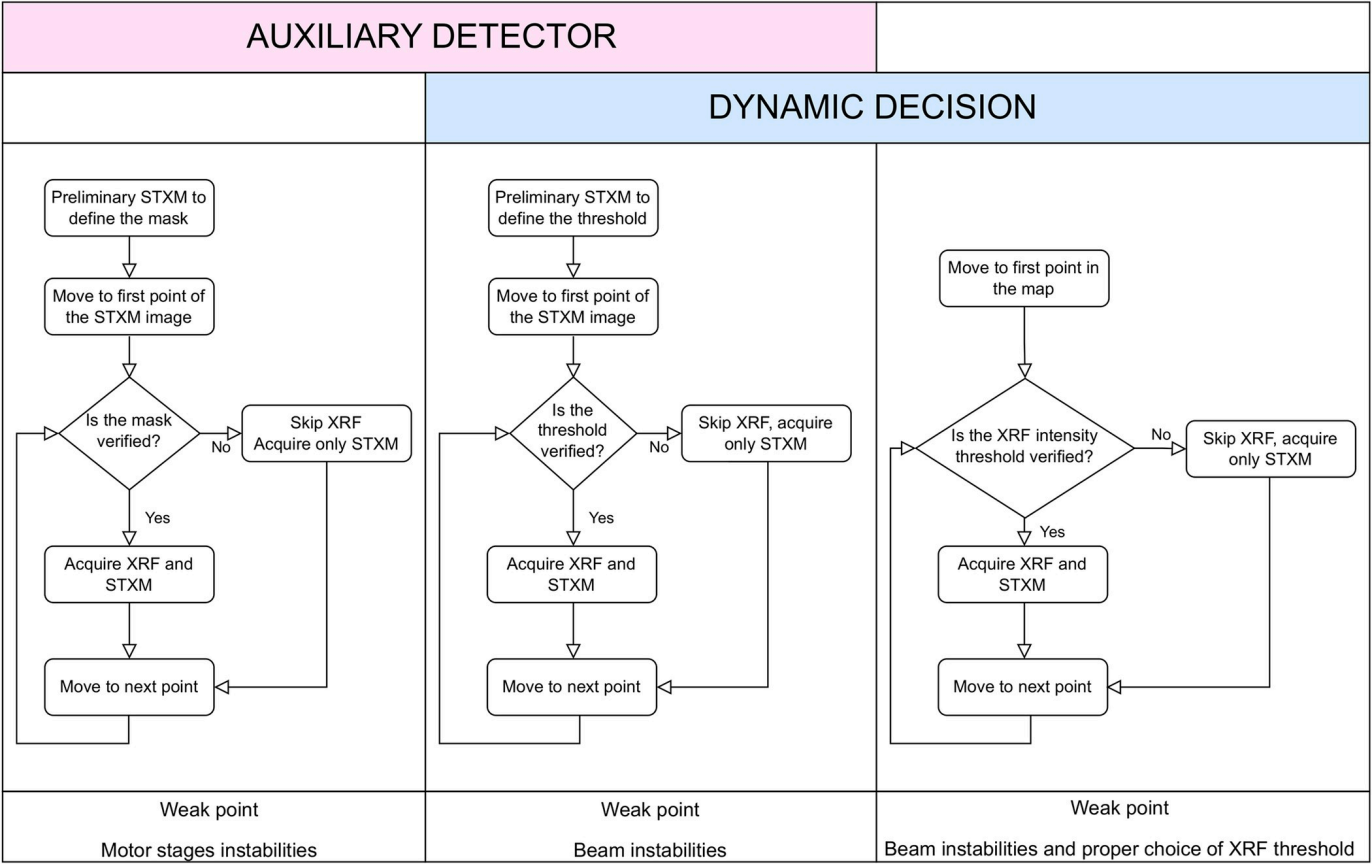
Current TwinMic CS methods. Schematic view of the three CS methods proposed in [5], highlighting the decision process workflow for each approach and their possible drawbacks.

The CS approaches shown in this manuscript are an upgrade to the original ones proposed in [5] and summarised in Fig 2. Two of the methods rely on the use of an auxiliary detector to determine whether or not to acquire the XRF signal. The first proposed one (Fig 2, column 1), is based on a static decision: as a first step a preliminary STXM image is acquired, not necessarily with the same resolution/step size of the final measurement, then a suitable mask is designed, in order to include only the interesting regions of the square/rectangular area. The mask is then converted into motor positions and uploaded to the control system. The XRF signal (together with STXM) will then be acquired only in the points where the mask is verified, while in the other points, only STXM signal will be collected, as it requires only a few milliseconds per pixel, compared to hundreds of milliseconds or even seconds for XRF. This approach also allows the acquisition of a few spare points outside the mask, in order to in-paint (filling/interpolating the sparser areas that were not scanned in detail) the non-mapped areas, for a better visualisation of the final results.

The second approach (Fig 2, column 2) is still based on the use of an auxiliary detector, the CCD camera, but in this case, it relies on a dynamic decision process. Again, a STXM preliminary map is acquired to inspect the sample, not necessarily with the same resolution/step size of the final scan, this time to define an absorption threshold which includes only the sample areas excluding the support. The predefined threshold is then inserted in the control software so that during the XRF acquisition, the control system will verify in real time and point by point, whether the absorption signal is higher than the preselected threshold; if it is, then the XRF signal will be acquired, otherwise only the STXM one will be collected.

The third method (Fig 2, column 3) is again based on a dynamic approach: for each point in the raster scan, the system will acquire a fast XRF signal and decide whether an element of interest is present or not, based on a predefined XRF intensity threshold usually evaluated from a ROI (Region Of Interest) in the spectra; from there, it will make the decision whether to further acquire a longer XRF signal or move to the next point. Note that we have recently developed an application to fit XRF spectroscopic data [18] which could be integrated into the presented system in order to carry out and verify the fitting in real time and point-by-point over the map, rather than evaluating ROIs of spectra.

A combination of the different methods can also be deployed [5]. For instance, the mask method can be combined with the absorption threshold one to quickly and accurately determine the ROIs.

In this work we illustrate an evolution of the first two above mentioned CS methods, that is, XRF mapping performed by exploiting the transmitted signals collected by the CCD camera.

### 2.1 Mask method with user friendly interface for direct user operation

**Fig 3:**
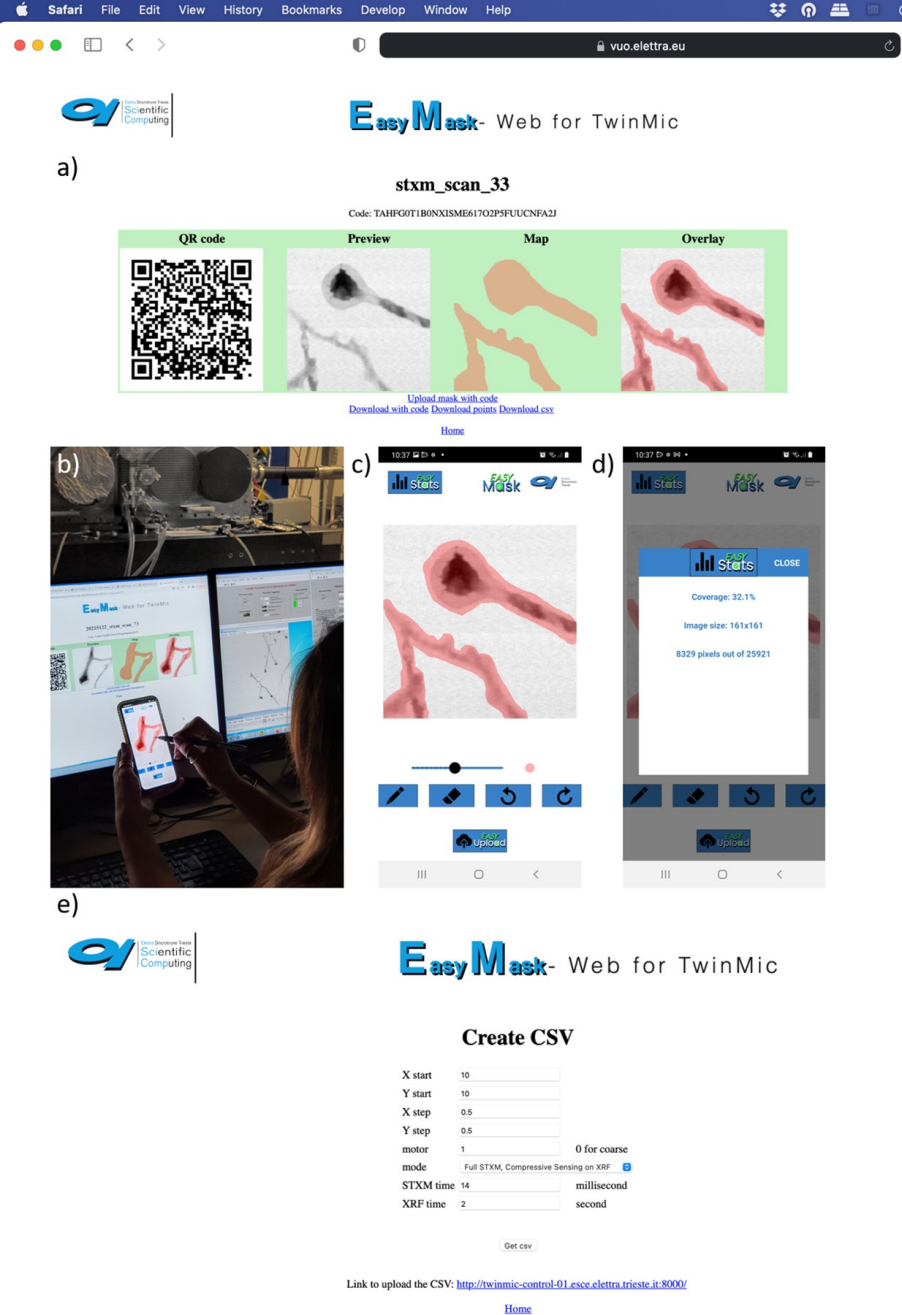
EasyMask application. EasyMask Application workflow, based on a web application (a) connected to a mobile one (b,c,d), generating a suitable scan path for both STXM and XRF acquisition (e).

The first method presented here is therefore based on the use of a mask, properly selected from a previously acquired STXM image. The STXM image does not need to be acquired with the same step size as the final XRF scan, as the mask can be properly upscaled to the correct size. Compared to our first proof of principle [5] (Fig 2, column 1) in this continuation work we further upgraded the acquisition software, called EasyMask, by making it more modern and user friendly. Specifically, we developed a mobile application which can be installed in Android OS, while the IOS version is in development. The STXM image is uploaded with a web application through our Virtual Unified Office (VUO) Elettra portal system which generates a QR code (QR code and Preview in Fig 3A). Once read through the mobile app, the image appears on the mobile screen (Fig 3B) and it can be easily edited using a pen. The drawn mask (Fig 3C) is then uploaded back to the web app (Map and Overlay panels in Fig 3A) and from there a suitable csv file can be generated, containing the acquisition parameters that will be read by the acquisition system (Fig 3E). The csv file contains the coordinates of the scan with the indication of the point where CS should be performed for XRF only or for both XRF and STXM. Additionally, acquisition times for STXM and XRF are inserted in the csv file. Once all the parameters are fixed, the csv file is uploaded by a web interface in the acquisition control system and the scan can be launched.

The EasyMask app also provides very useful statistical information highlighting the coverage percentage of the CS scan compared to the overall standard square/rectangular one (Fig 3D).

The introduction of such a modern and user-friendly interface, enables the non-expert user to actively participate in the experiments.

As already stated in [5], this approach may suffer from motor stage instabilities, as the mask is predefined based on the first fast STXM scan. In fact, as the XRF acquisition is much slower than the STXM, drifts may take place during the measurement. However state-of-the-art STXMs use modern sample stages that offer nanopositioning, encoders and interferometry, accurate enough for practical applications as the reproducibility of their positions is kept well below the typical scanning step size.

### 2.2 Dynamic threshold adjustment through real time diagnostics monitoring

The second method considered here is a more robust version of the one reported in [5] (Fig 2, column 2) and relies again on a preliminary STXM image collected from the area of interest, not necessarily with the same resolution/step size of the final measurement. An absorption threshold is selected from this overview STXM scan in order to cover only the area with the real sample. Indeed, if the specimen has an irregular shape and empty spaces, a standard square or rectangular acquisition scan would image a big portion of the sample support as well, which does not contain any useful scientific information. The threshold can be tuned in order to cover the interesting areas of the specimen and to cover regions with reasonable dimensions. Once selected and inserted in the TwinMic acquisition interface, the system will rapidly acquire a transmission signal from the whole area, while the slower XRF signal will be collected only where the absorption signal is higher than the pre-selected threshold. The system allows also the acquisition of the STXM signals only where the threshold is verified, but since the STXM acquisition time is just a few ms, for presentation reasons we often prefer to acquire the overall STXM. The described method is based on a dynamic choice during the scan, pixel by pixel, and of course implies X-ray beam stability in intensity and position, otherwise the pre-selected threshold cannot be the same for the whole scan area. Contrary to the mask method, it does not suffer from sample stage instabilities or drifts. Based on this observation and on the experience from our first proof of principle [5], we made the CS approach more robust by making the acquisition logic more advanced, that is, by adjusting the threshold in real time according to the beam fluctuations. This can be done by taking into account additional diagnostic values on beam position and other synchrotron parameters that can be used for real time normalisation and threshold adjustment, as described in the following paragraph. This solution allows us to overcome possible beam instabilities, which can happen especially for long acquisition scans.

#### 2.2.1 Multimodal acquisition including detectors of diagnostics

The techniques described in this manuscript are intrinsically multimodal; in the simplest form a fast detector workflow like that of STXM can be used to selectively trigger a slower detection process like that of XRF. A modern beamline endstation consists of additional detection systems that may not be used in a direct manner for imaging samples. An example of such a system is that of diagnostics and beam position monitoring detection. The TwinMic beamline is equipped with a beam position monitor (BPM) system composed of 4 blades at 90 degrees from one another [17], capable of providing data on beam fluctuations in intensity and/or position by measuring the current generated by the incoming beam on the blades themselves and by differentiating such currents among the blades. Such signals are then transmitted via a network (using TANGO, one of the main distributed control systems employed in large facilities [19]) and selectively stored in hdf5 files as metadata.

In particular, for our case, we can deploy and investigate three possible strategies: i) exploiting the information related to the accumulated ring current; ii) exploiting the signals collected on the BPM and iii) calculating in real-time the average signal of the last N pixels where N, by preliminary tests, should be twice the number of pixels/row, and then subtracting it from the current value. In S1 Fig we report a dataset consisting of the absorption image of a coronary artery from a rat [16], together with the ring current (S1B Fig) and the signals on the 4 blades (S1C–S1F Fig), recorded simultaneously during the scan. Note that, as demonstrated by panels a,b,c,d in S2 Fig, the signals collected on the BPM are affected by ring current, but they also contain other information, as they are sensitive to beam misalignment, not only to beam intensity changes. Indeed, after normalising the BPM signals by the ring current (S2A–S2D Fig), the ratio still shows some variations along the raster scan.

S3 Fig shows different normalisation approaches for the absorption image of S3A Fig, namely normalisation by the ring current (S3B Fig), normalisation by dynamic average calculation (S1C Fig), a combination of the previous approaches (S3D Fig) and nomalisation through the BPM signals. All of them successfully improve the original image (S3A Fig). The same approaches can be used for the differential phase contrast signals, as shown in S3D and S3G Fig.

All these solutions can be applied in real time during the scanning. The experiments performed demonstrated that such data from diagnostic detectors may be used to dynamically modulate the threshold that dynamically triggers the acquisition. Future implications of such direct involvement of diagnostic systems in the acquisition may impact the choice of hardware (i.e. frequency) and the communication of such data. It is expected that what is collectively referred to as diagnostics will be of importance for elaborate methods like those of this manuscript, since they can be used beyond the setup evaluation and impact in a direct way the actual data acquisition.

### 2.3 Dynamic XRF thresholding

This method is found to be the less efficient one in terms of speed as it relies on an acquisition of a first XRF spectrum, which usually requires an acquisition time longer than an absorption signal. However it can be strategically useful when other signals are not available, for instance when the samples are too thick to provide any transmission, but can still be mapped by XRF [20]. From a preliminary XRF map, an XRF threshold is identified by evaluating the XRF signal in a ROI corresponding to a specific chemical element of interest. In the final XRF map, the XRF will be acquired for a longer time only in the points where the threshold is verified. In the other point only a fast XRF spectrum will be collected and stored.

### 2.4 Machine learning

**Fig 4:**
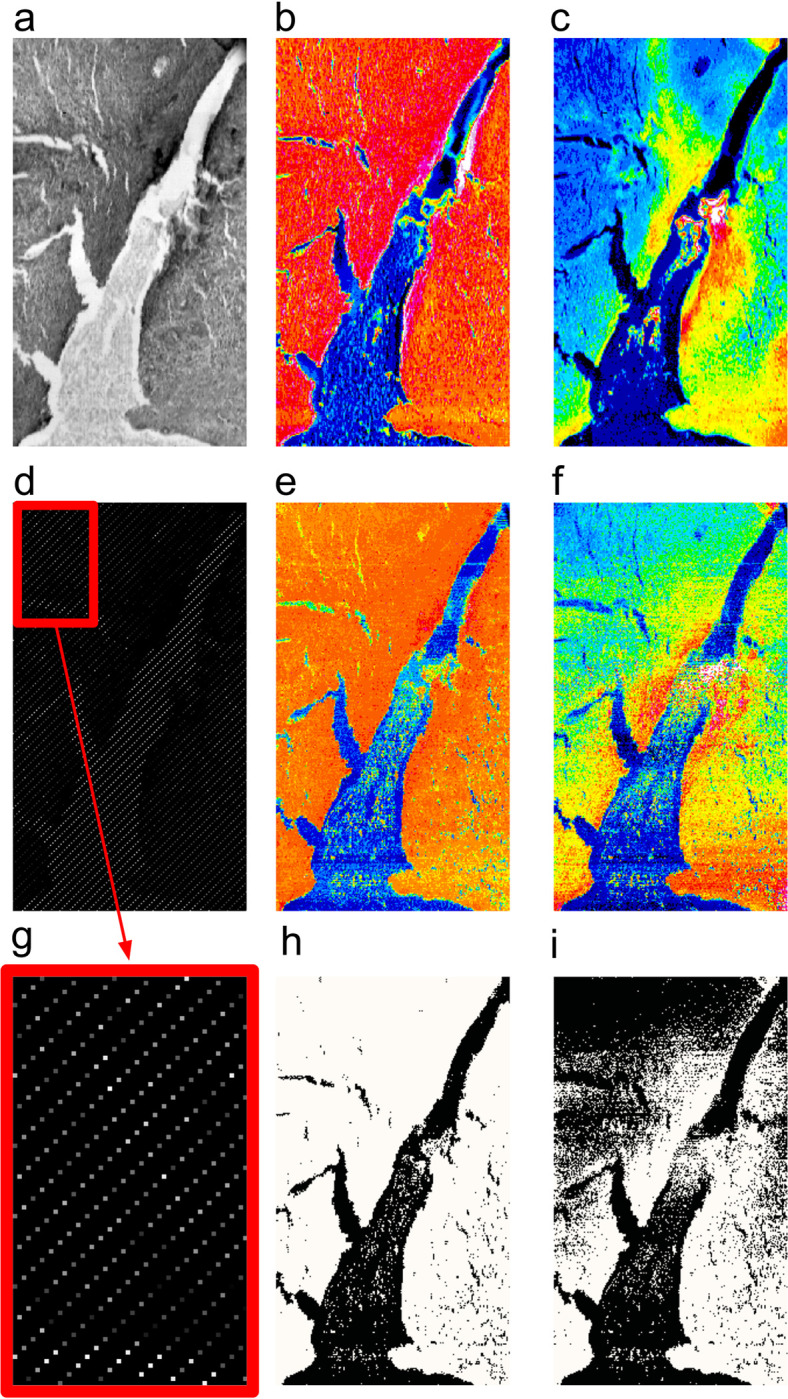
XRF map inferred through a ML approach. Standard STXM map (panel a) of Bovine Ovary tissue, accompanied by its O and Na XRF maps (panels b and c respectively). A ML model is trained as described in the text on the scan points marked in panel d (enlargement in panel g). For all the unseen positions, the model acts as an oracle, providing the corresponding XRF-like maps (panel e and f). The point-to-point threshold of the ML-generated maps are shown in panels h and i.

We are experimenting with ML for i) in-painting methods and ii) for deciding where to scan (full sampling) and where not to (CS-like sub-sampling). Our ML CS strategy can be outlined as: for a given sample (e.g. Bovine Ovary tissue [21]) i) acquire a standard/full STXM and a coarse/fast XRF map ii) train our neural network on transmission (TX) data (including differential phase contrast), diagnostics, in association with the XRF data. For subsequent samples (still the same or very similar setup conditions) we use the resulting trained model to infer XRF-like data, by processing point-by-point the corresponding STXM data. What we call ML generated XRF-like data inferred by only STXM ones are very limited and are still suitable enough to be used as a decisive factor (i.e. threshold) for acquiring real (and costlier) XRF data or not (see Fig 4).

In summary, the ML method we are introducing and testing for CS purposes is as follows: a new ML method for predicting the presence of an element has been developed. This method assumes a training step where it correlates TX+diagnostics with real XRF. This results in a system that guesses a trail XRF-like signal just from fast acquired TX+diagnostics. Subsequently this ML-generated XRF-like information (Fig 4E and 4F) is used as a clue to determine whether to trigger a real XRF acquisition for that scan point or not (Fig 4H and 4I), resulting in a CS reduced acquisition. Additional details are shown in S1 Appendix.

## 3. Results

### 3.1 Mask method

**Fig 5:**
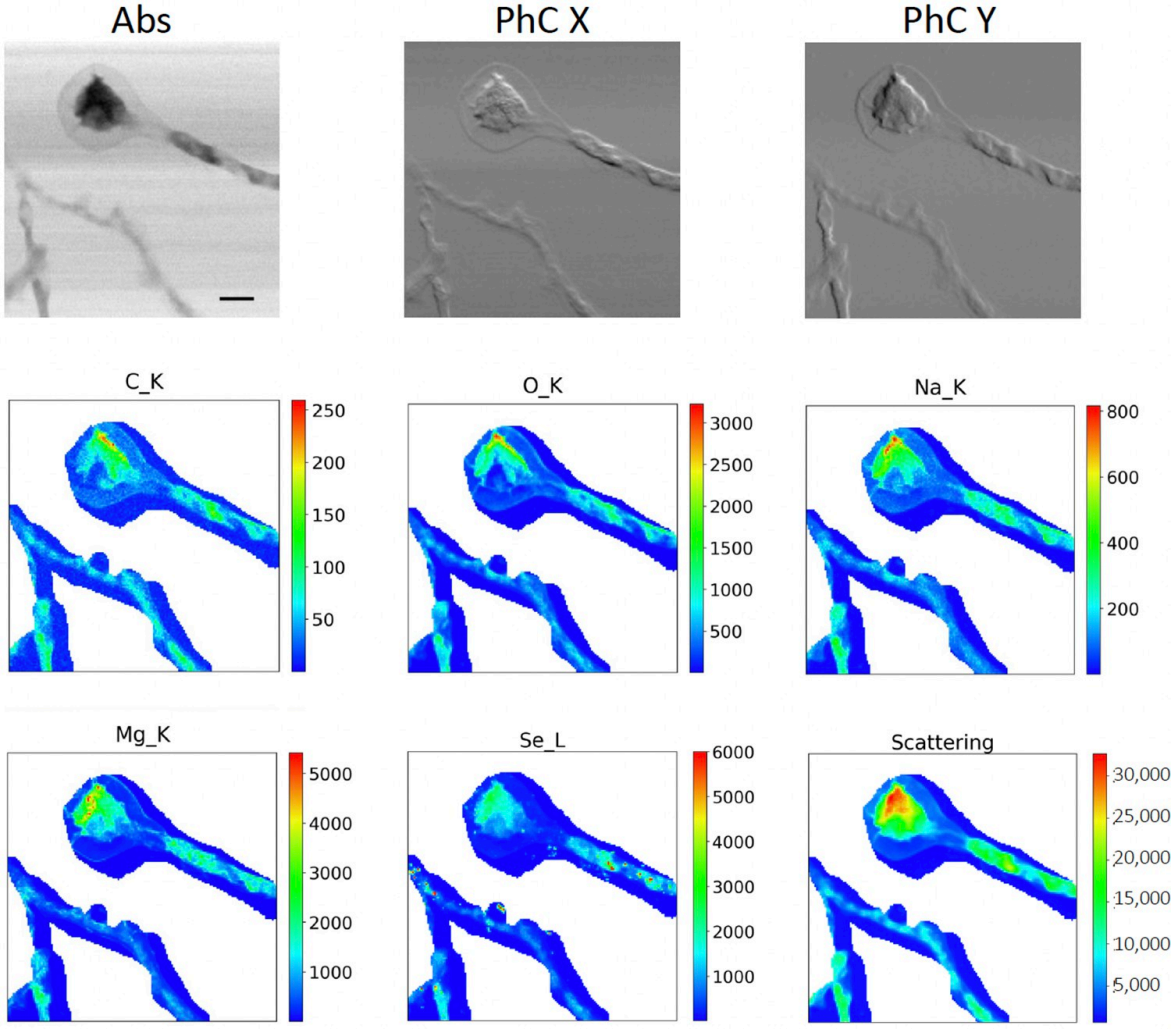
Example of EasyMask application. Absorption (Abs) and Differential Phase Contrast Images in X and Y (PhC X and PhC Y respectively) of the mycelium of the fungus Phycomyces blakesleeanus, with the corresponding masked XRF maps of C, O, Na, Mg, Se and Scattering, the latter acquired through the EasyMask mobile application. The scans were acquired in STXM mode with photon energy 1.75 keV with a spot size and a step size of 500 nm, over an area of 80 μm x 80 μm, with 2 s/pixel XRF acquisition time and 5 ms for STXM. Scale bar is 10 μm. The color bar units are counts.

Fig 5 depicts an example of a CS scan based on the use of the EasyMask mobile app for Android, where CS was applied on XRF only. It depicts the mycelium of the fungus Phycomyces blakesleeanus which has the ability to biotransform toxic selenium ions into non-toxic species and even to Se nanoparticles [22]. The corresponding average XRF spectrum acquired over the overall mapped area is shown in S4A Fig. A whole STXM scan was collected, while XRF signal was acquired only on the unmasked part, that is on the filaments, avoiding most of the empty Si3N4 window support. The XRF map can still be presented over a standard square or rectangular area.

Multiple variations are possible such as CS applied on STXM only, without acquiring any XRF or CS in both STXM and XRF.

The described approach is sensitive to motor or stage drifts, unless an interferometer or optical encoders’ reading correction system is applied to compensate for that. Since the method is based on a previously determined static condition, it cannot be adjusted or corrected in real time, but a combination of the different described methods is also possible. Indeed a wider mask could be applied to compensate for possible drifts, and inside that a threshold decision (method 2 in Fig 2), to remove unwanted areas, similarly to what is shown in [5]. This would decouple the success of the scan from stage instabilities or shifts.

The novelty of EasyMask relies on its seamless interaction with STXM data that are present on the facility’s central data storage, while the app is not a control system per se, but an auxiliary input instrument (for example it allows for the use of pens). The app is coupled with a web app present in the VUO, where STXM images are coupled with QR codes; when the QR codes are scanned by the app, the STXM image appears on the smartphone. The QR contains suitable information for uploading the generated mask and associating it with its corresponding scan positions. Those positions are subsequently used by the acquisition system for performing sparse scans as part of compressive sensing experiments as first developed in Elettra.

Each component of the presented workflow is inspired by previous systems but is re-designed for current and future challenges like remote operation, FAIR data, and demanding computational analysis. The present state is that of a prototype in continuous development that has demonstrated its validity, usefulness and feasibility.

Note that the VUO system is a portal where all Elettra Sincrotrone Trieste external users and personnel must be registered to submit proposals, access data and obtain access permission to the facility, among other things.

### 3.2 Absorption threshold method

A typical application field of STXM coupled with XRF is environmental science and specifically plant biology, where it may be important to determine the distribution of a specific chemical element in leaves, flowers and/or roots. Such an element may be of interest to understand the plant’s optical properties [23], or for a better understanding of plant uptake from the soil [24], or may cause toxicity and damage to the plants themselves [25–27] or even for phytoremediation purposes, where plants are used to reclaim contaminated lands, as they can absorb toxic compounds from the soil and transform them into harmless ones [28, 29]. Plant tissues such as leaves, roots, flowers or roots sections usually have irregular shapes and are highly porous, thus rectangular or square scans acquire signals also on empty or on uninteresting areas. Hence CS approaches are particularly useful for such specimens.

**Fig 6:**
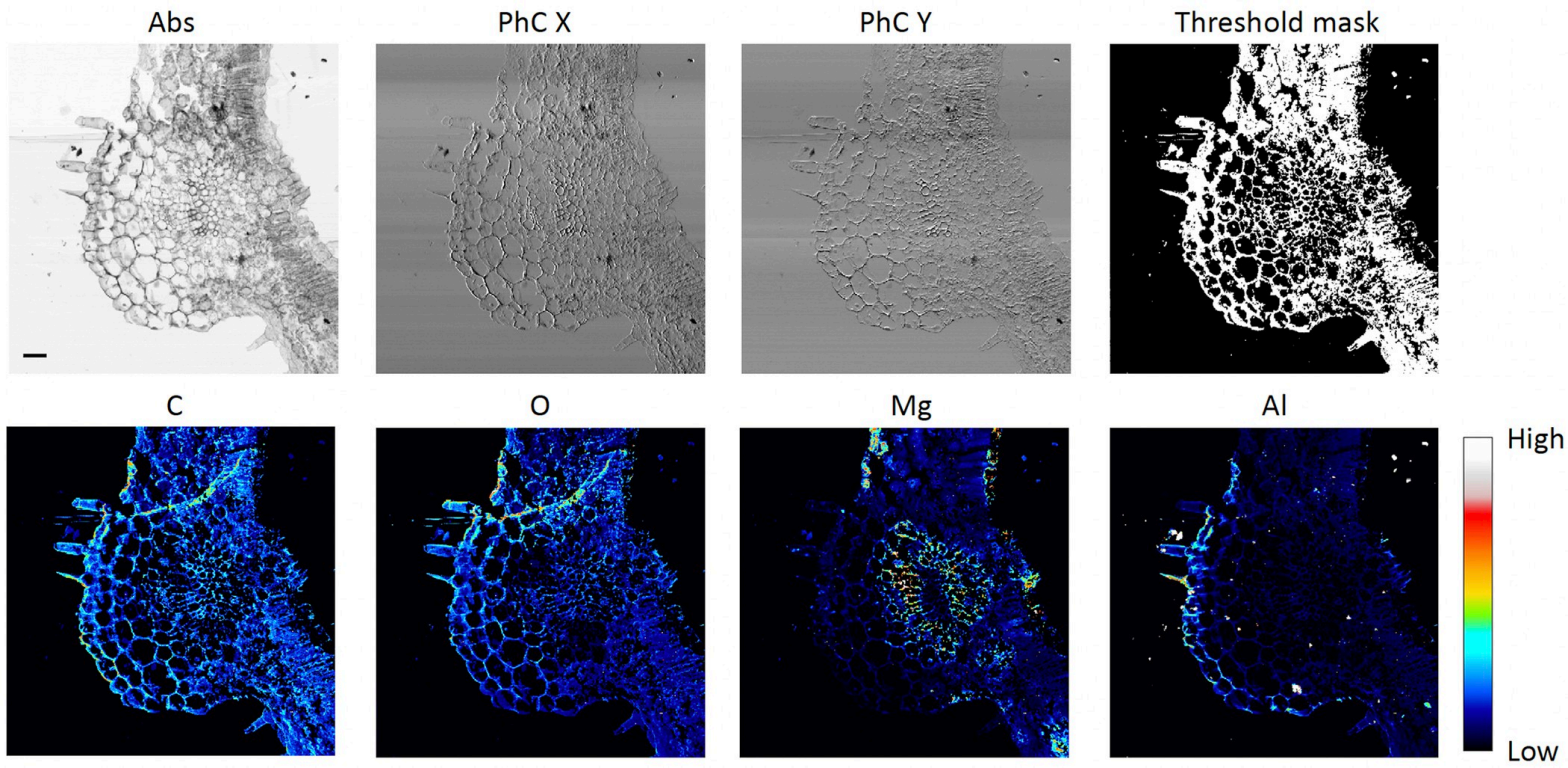
Absorption threshold with dynamic correction. Upper row: Absorption (Abs) and Differential Phase Contrast Images in X and Y (PhC X and PhC Y respectively) of a section of tomato leaf. Lower row: corresponding XRF maps of C, O, Mg and Al, obtained with the threshold method by acquiring the XRF signal only on the white pixels of the panel Threshold Mask. The scans were acquired in STXM mode at photon energy 2 keV with a spot size and a step size of 1 μm, over an area of 700 μm x 700 μm, with 1 s/pixel XRF acquisition time and 5 ms for STXM. Scale bar is 50 μm. All images were normalised by incident flux.

Fig 6 shows an example of the threshold approach. It depicts a section of a tomato leaf. Also, in this case a preliminary STXM image was acquired to determine the absorption threshold. During the final scan the STXM signal was acquired everywhere, while the XRF one was collected only on the points with absorption higher than the pre-selected threshold. Clearly the scientific information is retained while the CS method allows a saving of 70% of the time and of the storage space, as only 30% of the total number of pixels was acquired in LEXRF mode. For the present dataset a regular scan would have required 164 hours, while, by applying the described CS methods, it needed 48 h. During the acquisition a threshold mask (Threshold Mask Panel in Fig 6) is then created and saved as a record. Again, the XRF signals can be presented in a standard way, through a square or rectangular representation, even if not acquired everywhere. The corresponding average XRF spectrum acquired over the overall mapped area is shown in S4B Fig.

The savings in time can be initially estimated, and later on calculated, by evaluating the ratio between the area covered with the selected threshold and the overall area in the preliminary and in the final STXM respectively. That gain depends on the sample shape and can vary from situation to situation. Consequently, the user should evaluate case by case if the CS methods can provide an advantage compared to standard square or rectangular scans. Usually most irregularly shaped specimens can benefit significantly from CS methods.

Contrary to the mask method, the absorption threshold one does not suffer from motor stages’ instabilities; however, it is sensitive to beam instabilities, if not properly accounted for, since the threshold is predetermined, beam intensity changes may alter the portion of scan to be acquired. However, being based on a dynamic decision process, point-by-point in the raster scan, smart correction can be applied in real time. Indeed, the raw acquired data may be affected by beam instabilities, as shown in S5 Fig which depicts the original absorption data; however additional diagnostic systems can allow dynamic thresholding in real time, and successfully provide a double benefit: i) maintaining the desired masked area and ii) obtaining a way to normalise the STXM and XRF acquired data, as shown in the final images depicted in Fig 6.

### 3.3 Dynamic XRF thresholding method

As described earlier in paragraph 2.3, even in advanced setups where multimodal acquisitions like STXM+XRF are available, there are cases where the fast probe (e.g. STXM) cannot be used. A sample may not transmit while XRF emission is still possible. The lack of STXM required by methods 1 or 2 (Fig 2) can be overcome through suitable real time XRF signal evaluation at fast acquisition times (e.g. 0.1 s instead of 3 s). In this case the resulting CS scan has variable XRF acquisition times. In the simplest of cases an element that is known to be in high concentration in a region of interest (e.g. O in a cell or Si in stone) is used as the deciding factor for a longer acquisition in the hope of identifying elements that are both present on those areas that cannot be revealed at lower acquisition times (e.g. Mg in cell or Na in a stone). In simpler terms, the system assumes that there is no reason to check for Mg in areas where O is not present, that is outside the cell, or for Na in a stone where Si has not been revealed. More complex yet feasible applications of this XRF-only CS method require seeking the “signature of an element” which may be a specific type of XRF spectrum that when is correlated with that of a fast acquisition instructs for a longer acquisition on that point (e.g. better statistics for given elements of interest) [5]. Prior calibrations and the use of standards on specific beamline setups, through suitable PCA and clustering techniques, demonstrated that the extraction of such signatures is feasible.

**Fig 7:**
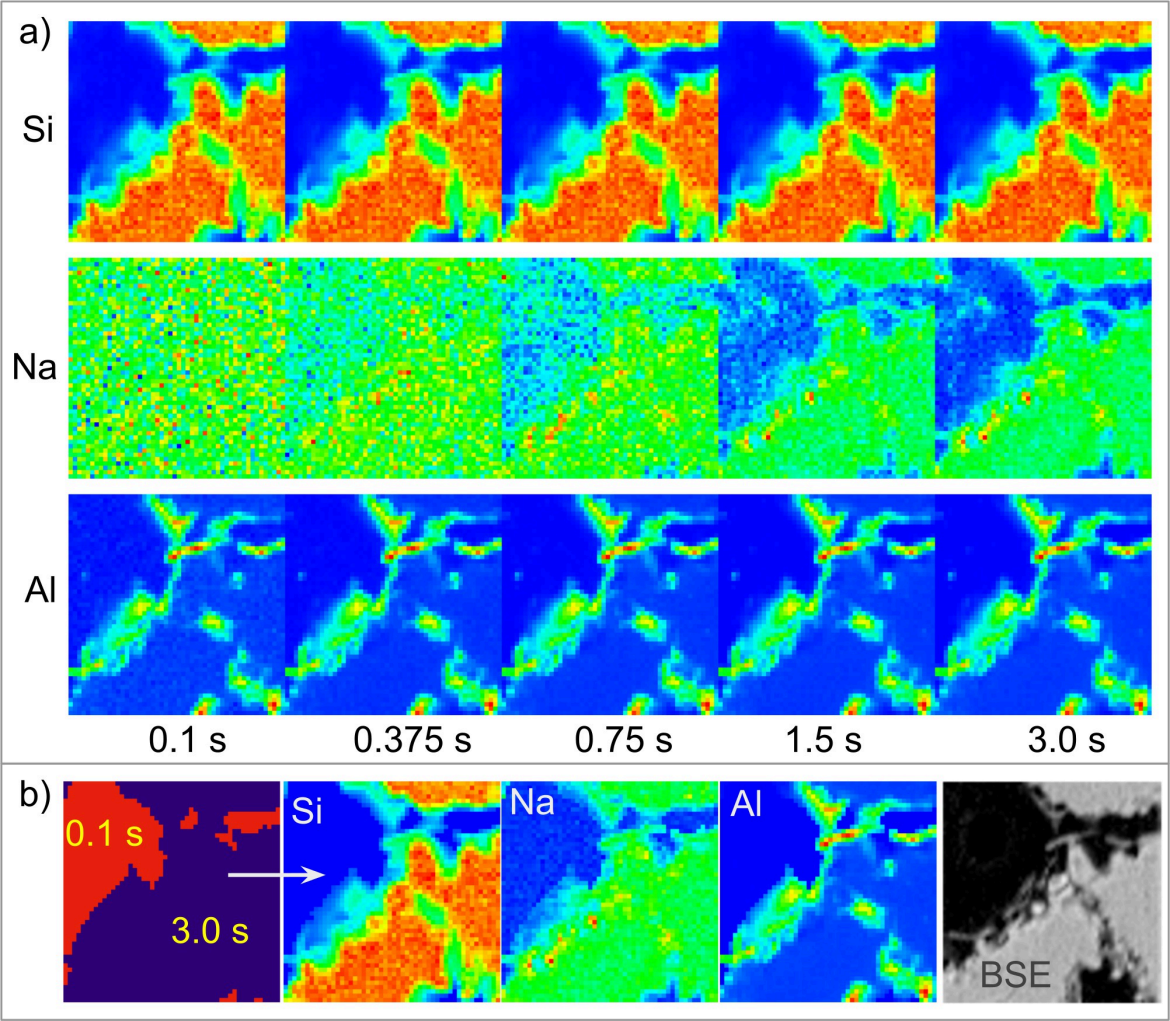
XRF-only dynamic scan. XRF-only dynamic CS scans. A) XRF maps of Si, Na and Al acquired at 0.1 s, 0.375 s, 0.75 s, 1.5 s and 3 s on the non-transmitting sandstone sample shown in panel BSE (Back Scattered Electron signal), panel b, demonstrating that XRF at fast 0.1 s acquisition is enough to reveal Si and Al, but not enough for Na which required more than 1.5 s. The rapidly acquired Si signal, evaluated in real-time and point-by-point, produces a threshold which determines where the map is acquired at 0.1 s and at 3.0 s (first image in panel b) respectively. The resulting 25% reduced scan (Si, Na and Al XRF maps in panel b) achieves high XRF counts in 3 s on the regions of interest while underexposing the rest. The XRF maps were acquired at the TwinMic beamline at 2 keV excitation energy, spot size and 1.6 μm step size.

Fig 7 shows an example of dynamic XRF-only thresholding strategy on a 1 mm thick sandstone exposed to a nano-protective product [20]. The sample’s thickness and composition does not allow transmission (necessary for STXM), thus previously collected visible light and Backscattered Electron (BSE) (panel BSE in Fig 7B) images were used to navigate on the surface of the sample. The aim of the research was to determine the presence of specific chemical elements, related to the nano-protective compound, in a silicate-based stone, where holes are also present and ideally do not need to be scanned. By applying an XRF threshold on Si, the holes were skipped. In this specific case several XRF maps were collected at different acquisition times (0.1, 0.375, 0.75, 1.5 and 3 s), visible in Fig 6, to investigate the XRF signal on a trace element such as Na, and establish the lowest possible acquisition time providing a suitable threshold. Then the XRF threshold was applied, producing XRF maps of Si, Na and Al with variable times (0.1 s on less interesting areas and 3 s for the main sample), as shown in Fig 7B, allowing a saving of approximately 25% of the total time. For the present dataset a regular scan would have required 1.5 hours at 3 s per pixel acquisition time, while, by applying the described CS methods, it needed around 20 minutes. Once the system is calibrated (i.e. the threshold and acquisition times are established), this can be applied to all areas/samples of the same type, in this case.

### 3.4 Machine learning approach

A demonstration of the ML approach described in paragraph 2.4 is reported in Fig 4, where an XRF-like map inferred by ML is compared with a real XRF one acquired on a 5 μm thick paraffined slice of bovine ovary tissue, obtained from a young heifer at a slaughterhouse (macello Pelizzari, Loria, Treviso, Italy). More details on the sample preparation are given in [21].

A ML model is trained as described in paragraph 2.4 on the scan points marked in panel d. For all the unseen positions, the model acts as what in ML terms is described as an oracle, predicting the corresponding ML XRF-like maps (panel e and f) which closely resemble the content of panel b and c. The point-to-point threshold of the ML-generated maps (Fig 4, panels h and i) demonstrates the feasibility of the technique in real case studies.

## 4. Discussion

In this paper we report robust and user-friendly versions of compressive sensing methods for XRF spectromicroscopy, whose principle could also be applied to other types of imaging or microscopy. While in previous works our team focused only on the methodology, in this manuscript we introduce a new technological development in the form of a mobile app, which is employed as an input device for the acquisition system. The main goal and wish behind such developments, is to promote the widespread use of CS techniques in the field. We expect that CS approaches will become more and more widespread thanks to their efficacy and powerful outcomes.

The authors are currently exploring the application of CS solutions in ptychography imaging, reducing unnecessary data collection and improving the time efficiency of the acquisition.

Last but not least on the methodological frontier, special attention is directed to the conditional clauses, which represent the logic establishing on-the-fly how an acquisition should take place. Various techniques have been proposed in recent years, mostly based on real-time signal evaluation from one or more detectors and modalities. Our team is currently focusing on the advanced application of Machine Learning methods for their inclusion in the decision process of CS.

## Supporting information

S1 Fig a) Absorption images of a coronary artery rat section acquired at a 1.5 keV excitation energy with a 2.5 μm step size, over an area of 2100 μm × 2000 μm; b) storage ring current trend during the acquisition of the STXM image of panel a; current signals (c-f) recorded point by point of the raster scan on the 4 BPM blades present at the entrance of the TwinMic beamline.
(DOCX)

S2 Fig a-d Current signals recorded point by point of the raster scan on the 4 BPM blades (S1C–S1F Fig) present at the entrance of the TwinMic beamline, each normalised point-by-point by the storage ring current (S1B Fig) recorded simultaneously.
(DOCX)

S3 Fig a) original raw STXM absorption image of the coronary artery section and b) corresponding STXM image normalised by the ring current, c) normalised by using the floating window average normalisation, d) normalised by using both ring current and floating window average and e) normalised using the BPM signals. f) Raw STXM differential phase contrast in X image of the coronary section and g) corresponding STXM differential phase contrast in X image normalised using both ring current and floating window average normalisation.
(DOCX)

S4 Fig Average XRF spectrum of the areas mapped in Fig 5 and Fig 6 with the indication of the detected XRF emission lines.
(DOCX)

S5 Fig Original transmission image. Original raw transmission image of the tomato leave, as acquired during the XRF scan depicted in Fig 6. By real time correction of the threshold, the desired mask is preserved and the final images can be also normalised, as shown in Fig 6.
(DOCX)

S1 Appendix Neural Network details for ML based-method.
(DOCX)
