# Supplementary material for: Advances in sparse dynamic scanning in spectromicroscopy through compressive sensing
Source: PLoS One. 2023 Nov 9;18(11):e0285057. doi: 10.1371/journal.pone.0285057 (PMC10635485; doi:10.1371/journal.pone.0285057)

**Figure S3.** a) original raw STXM absorption image of the coronary artery section and b) corresponding STXM image normalised by the ring current, c) normalised by using the floating window average normalisation, d) normalised by using both ring current and floating window average and e) normalised using the BPM signals. f) Raw STXM differential phase contrast in X image of the coronary section and g) corresponding STXM differential phase contrast in X image normalised using both ring current and floating window average normalisation.


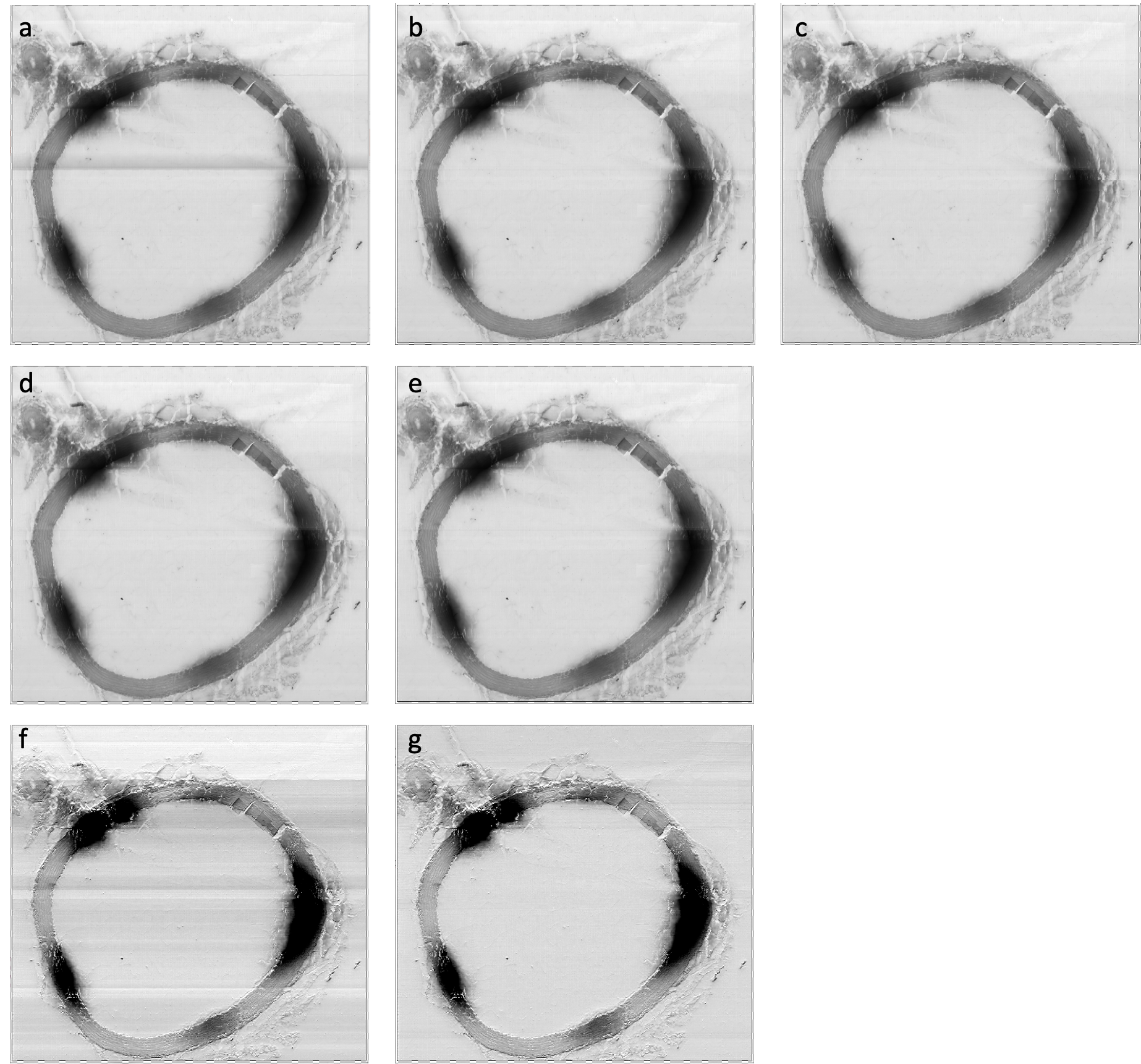

Supplement: S3 Fig — a) original raw STXM absorption image of the coronary artery section and b) corresponding STXM image normalised by the ring current, c) normalised by using the floating window average normalisation, d) normalised by using both ring current and floating window average and e) normalised using the BPM signals. f) Raw STXM differential phase contrast in X image of the coronary section and g) corresponding STXM differential phase contrast in X image normalised using both ring current and floating window average normalisation. (DOCX) [file pone.0285057.s003.docx]
