# Supplementary material for: Advances in sparse dynamic scanning in spectromicroscopy through compressive sensing
Source: PLoS One. 2023 Nov 9;18(11):e0285057. doi: 10.1371/journal.pone.0285057 (PMC10635485; doi:10.1371/journal.pone.0285057)

**Figure S4.** Average XRF spectrum of the areas mapped in Figure 5 (a) and Figure 5 (b) with the indication of the detected XRF emission lines.


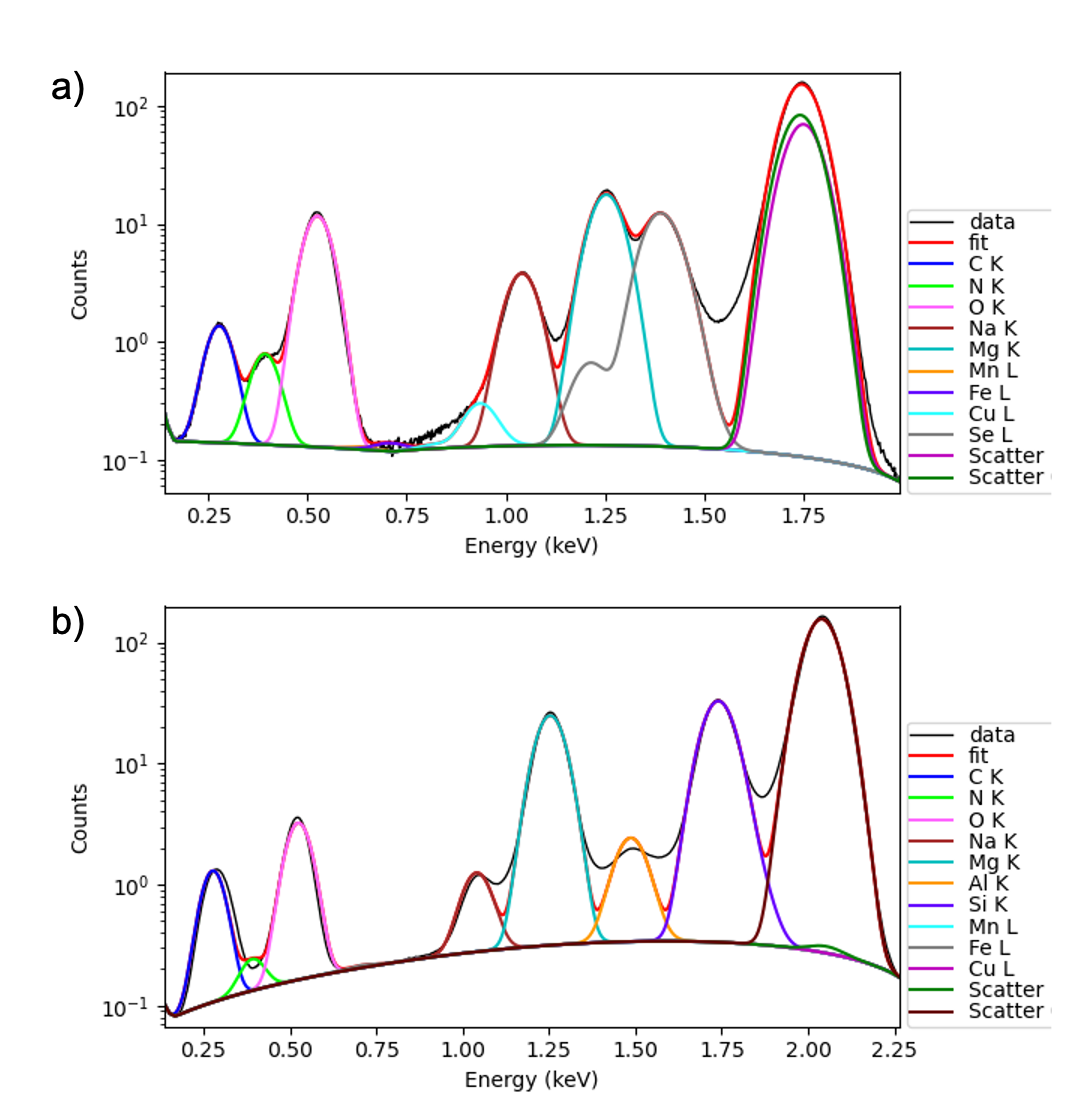

Supplement: S4 Fig — Average XRF spectrum of the areas mapped in Fig 5 and Fig 6 with the indication of the detected XRF emission lines. (DOCX) [file pone.0285057.s004.docx]
