# Supplementary material for: Advances in sparse dynamic scanning in spectromicroscopy through compressive sensing
Source: PLoS One. 2023 Nov 9;18(11):e0285057. doi: 10.1371/journal.pone.0285057 (PMC10635485; doi:10.1371/journal.pone.0285057)

**Figure S5.** Original raw transmission image of the tomato leaf, as acquired during the XRF scan depicted in Figure 6. By real time correction of the threshold, the desired mask is preserved and the final images can be also normalised, as shown in Figure 6.


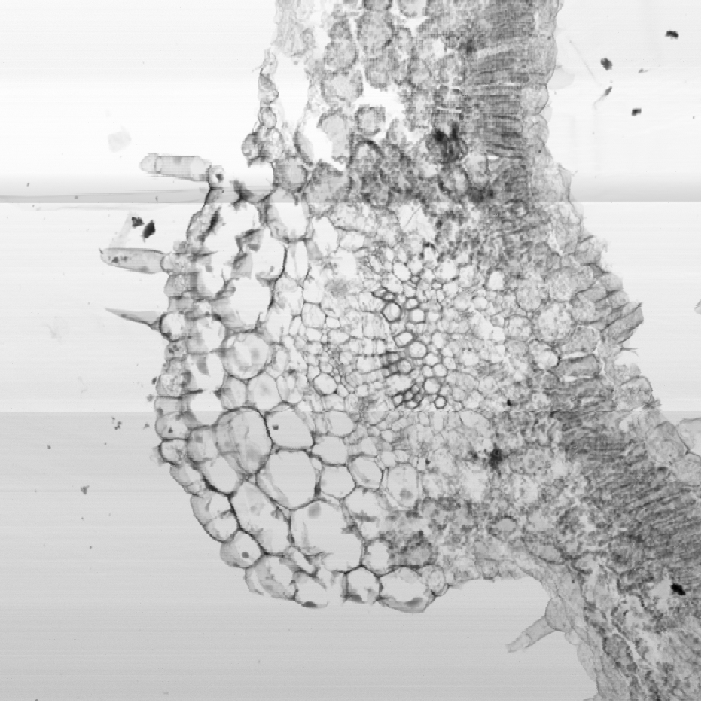

Supplement: S5 Fig — Original raw transmission image of the tomato leave, as acquired during the XRF scan depicted in Fig 6. By real time correction of the threshold, the desired mask is preserved and the final images can be also normalised, as shown in Fig 6. (DOCX) [file pone.0285057.s005.docx]
