# Supplementary material for: Advances in sparse dynamic scanning in spectromicroscopy through compressive sensing
Source: PLoS One. 2023 Nov 9;18(11):e0285057. doi: 10.1371/journal.pone.0285057 (PMC10635485; doi:10.1371/journal.pone.0285057)
Supplement: S1 Appendix — Neural Network details for ML based-method. (DOCX) [file pone.0285057.s006.docx]

**S1 Appendix: Neural Network details for ML based-method**

Additional details in regard to the realisation of our ML method include the use of supervised learning as the training strategy: for each ith scan point, the network is trained to generate a vector of 1024 points - a spectrum - by observing only a vector of 9 features, namely {absorption, differential phase contrast in x and y, phase, storage ring current, BPM readings on the 4 blades}; the first four derive from calculations on the transmission detector data (intensity value, differential phase contrast for x and y, integrated phase [S1]), while the remainings are diagnostic values retrieved by the storage ring control systems (ring current and beam position monitor readings). In the training scheme, the acquired XRF spectrum represents indeed the ground truth - the labels - that the network is trained to mimic, conditioned by the status of its internal trained parameters and the input vector. The Neural Network (NN) is designed as a bottleneck structure [S2], made by cascading 5 densely connected layers with PReLU [S3] non linear activation. Batch normalisation layers are interleaved between any representational layer to reduce the risk of overfitting. Sigmoid non linear activation is used for the output nodes. The total amount of trainable parameters is then roughly 230000.

[S1] M. D. de Jonge, B. Hornberger, C. Holzner, D. Legnini, D. Paterson, I. McNulty, C. Jacobsen, and S. Vogt, Phys. Rev. Lett. 100, 163902 – Published 22 April 2008

[S2] LeCun, Y., Bengio, Y. & Hinton, G. Deep learning. Nature 521, 436–444 (2015). https://doi.org/10.1038/nature14539

[S3] K. He, X. Zhang, S. Ren and J. Sun, "Delving Deep into Rectifiers: Surpassing Human-Level Performance on ImageNet Classification," 2015 IEEE International Conference on Computer Vision (ICCV), Santiago, Chile, 2015, pp. 1026-1034, doi: 10.1109/ICCV.2015.123.
