# Supplementary material for: Advances in sparse dynamic scanning in spectromicroscopy through compressive sensing
Source: PLoS One. 2023 Nov 9;18(11):e0285057. doi: 10.1371/journal.pone.0285057 (PMC10635485; doi:10.1371/journal.pone.0285057)
Supplement: S1 Fig — a) Absorption images of a coronary artery rat section acquired at a 1.5 keV excitation energy with a 2.5 μm step size, over an area of 2100 μm × 2000 μm; b) storage ring current trend during the acquisition of the STXM image of panel a; current signals (c-f) recorded point by point of the raster scan on the 4 BPM blades present at the entrance of the TwinMic beamline. (DOCX) [file pone.0285057.s001.docx]

**Figure S1.** a) Absorption images of a coronary artery rat section acquired at a 1.5 keV excitation energy with a 2.5 µm step size, over an area of 2100 µm × 2000 µm; b) storage ring current trend during the acquisition of the STXM image of panel a; current signals (c,d,e,f) recorded point by point of the raster scan on the 4 BPM blades present at the entrance of the TwinMic beamline.
