# Supplementary material for: Advances in sparse dynamic scanning in spectromicroscopy through compressive sensing
Source: PLoS One. 2023 Nov 9;18(11):e0285057. doi: 10.1371/journal.pone.0285057 (PMC10635485; doi:10.1371/journal.pone.0285057)
Supplement: S2 Fig — a-d Current signals recorded point by point of the raster scan on the 4 BPM blades (S1C–S1F Fig) present at the entrance of the TwinMic beamline, each normalised point-by-point by the storage ring current (S1B Fig) recorded simultaneously. (DOCX) [file pone.0285057.s002.docx]

**Figure S2.** a,b,c,d Current signals recorded point by point of the raster scan on the 4 BPM blades (Figure S1c,d,e,f) present at the entrance of the TwinMic beamline, each normalised point-by-point by the storage ring current (S1_Fig b) recorded simultaneously.
